# A tutorial on capturing mental representations through drawing and crowd-sourced scoring

**DOI:** 10.3758/s13428-021-01672-9

**Published:** 2021-08-02

**Authors:** Wilma A. Bainbridge

**Affiliations:** Department of Psychology, University of Chicago, 5848 S University Ave, Beecher Hall 303, Chicago, IL 60637 USA

**Keywords:** Visual production, Mental representations, Computer vision, Online experiments

## Abstract

When we draw, we are depicting a rich mental representation reflecting a memory, percept, schema, imagination, or feeling. In spite of the abundance of data created by drawings, drawings are rarely used as an output measure in the field of psychology, due to concerns about their large variance and their difficulty of quantification. However, recent work leveraging pen-tracking, computer vision, and online crowd-sourcing has revealed new ways to capture and objectively quantify drawings, to answer a wide range of questions across fields of psychology. Here, I present a tutorial on modern methods for drawing experiments, ranging from how to quantify pen-and-paper type studies, up to how to administer a fully closed-loop online experiment. I go through the concrete steps of designing a drawing experiment, recording drawings, and objectively quantifying them through online crowd-sourcing and computer vision methods. Included with this tutorial are code examples at different levels of complexity and tutorials designed to teach basic lessons about web architecture and be useful regardless of skill level. I also discuss key methodological points of consideration, and provide a series of potential jumping points for drawing studies across fields in psychology. I hope this tutorial will arm more researchers with the skills to capture these naturalistic snapshots of a mental image.

## Introduction

A key goal of psychological research is to understand the mind and brain through observations of behavior. These behavioral observations are often limited to low-resolution outputs—such as reaction time and choice—largely because of their ease of quantification and clear links to several cognitive processes. However, there are fundamental questions that cannot be answered by single-value outputs, such as the content of one’s mental representations for an item. Here, I will demonstrate how *drawing* as a high-dimensional behavioral output can be utilized to reveal new insights about human cognition. Although drawings may seem subjective and highly variable, recent techniques from Big Data and citizen science have made objective quantification of drawings at a large scale accessible to psychologists across the field.

Historically, drawings have had relatively limited usage as a behavioral output in psychology. Drawings of simple objects or abstract shapes have been used clinically, to diagnose memory disorders as part of the Wechsler Memory Scale test battery (Wechsler, 2009), to quantify spatial neglect (Agrell & Dehlin, 1998), and to investigate parietal cortex lesions (Makuuchi et al., 2003). Outside of clinical diagnosis, a high proportion of drawing studies focus on examining the drawings of children as insight into underlying thoughts or feelings, often towards a psychoanalytic or therapeutic aim (e.g., Kosslyn et al., 1977; Otgaar et al., 2016; Thomas & Jolley, 1998). However, the potential reach of drawing research goes far beyond diagnostic or therapeutic applications; drawing also holds promise as a basic research method that can reveal general principles about cognition across ages. Psychology research in the 1980s and 1990s occasionally utilized drawing as a measure, for example, to understand the schemas of familiar objects (Rubin & Kontis, 1983), or to quantify memory for scene boundaries (Inraub & Bodamer, 1993). However, as computers became more popular tools for administering experiments and recording behavior (e.g., reaction times), drawing as a behavioral measure became less common. Many researchers grappled with the subjective nature of drawings, and utilized small and simple stimulus sets along with basic drawing measures to limit variability in drawing behavior.

Such variability can instead be leveraged to gain rich insight into a wide range of cognitive processes reflective of how people view, prioritize, remember, and interpret both external information and internal representations. One can easily transform drawings into a large set of measures by combining high numbers of participants per image, principles from computer vision, and objective, crowd-sourced quantification. These measures can capture varied information including object detail, spatial accuracy, or errors. In a recent study, drawings of real-world photographs revealed an unprecedented richness and accuracy to visual memory, with participants drawing from memory an average of 150 objects across 12 scenes, in pixel-precise locations, with few false memories (Bainbridge et al., 2019). This level of performance reached much beyond the prediction of nine items at maximum that would be made by verbal recall memory studies (Murdock, 1962). In fact, drawing as a mnemonic strategy has been shown to outperform verbally based, imagery-based, and semantic elaborative strategies (Wammes et al., 2016; Wammes et al., 2019), even for memory of highly abstract concepts (Roberts & Wammes, 2020). Furthermore, learning to draw may boost one’s ability to efficiently perceive and encode visual information (Perdreau & Cavanagh, 2014; Perdreau & Cavanagh, 2016; Vogt & Magnussen, 2007). Computer vision-based metrics such as saliency models and meaning maps (Henderson & Hayes, 2017) are also able to significantly predict the objects that are drawn from memory of a scene (Bainbridge et al., 2019), revealing a new potential for utilizing computational models to make precise, content-based predictions of memory.

We have continued to explore a wide range of important cognitive questions using drawings to reveal the detail within memory. We used scene drawings to reveal evidence against the classic textbook phenomenon of *boundary extension*—a propensity to extrapolate beyond the borders of a photograph, said to reflect our mind’s propensity to automatically “fill in” information. By investigating drawings made from a diverse range of scenes mirroring the statistics of the real world, we found the opposite effect of boundary contraction was just as likely (Bainbridge & Baker, 2020a), and this work has ignited a new debate in the fields of scene perception and memory (Bainbridge & Baker, 2020b; Intraub, 2020; Park et al., 2021). In another study, drawings from participants with *aphantasia*—a newly identified condition characterized by a lack of visual imagery (Jacobs et al., 2018; Keogh & Pearson, 2018; Zeman et al., 2015)—revealed a specific deficit in object memory over spatial memory, suggesting separate systems to support visual imagery (Bainbridge, Pounder, et al., 2021a). This study has motivated follow-up neuroimaging research on differences between perception and imagery (Bainbridge et al., 2021c). We have also revealed how semantic consistency (Bainbridge, Kwok, & Baker, 2021b) and categorical competition (Hall et al., 2021) influence distortions in memory drawings. While these examples largely deal with topics related to perception and memory, these drawing methods can easily be applied to other topics, such as attention, emotion, morality, conceptual thinking, decision-making, and social psychology. Drawings could also be used to characterize mental representations in unique populations, such as children, older adults, and those with conditions that may influence percepts, concepts, or memories, such as autism spectrum disorder, schizophrenia, or Alzheimer’s disease.

This tutorial will describe how researchers can implement their own drawing-based experiments, and how they can score these drawings through online crowd-sourcing methods. I provide examples of how these methods can be combined with other methods such as computer vision or machine learning to add a predictive component to this research. This tutorial also includes a publicly available code base and tutorials from which researchers can build their experiments, even with limited programming experience (https://osf.io/tgavx/). In a time when online research is becoming increasingly important and popular, these tutorials have been designed to teach basic principles of web architecture and online research, so that experimenters will become more comfortable thinking about online data collection more broadly, in this framework of designing a drawing experiment.

## The bipartite structure of a drawing study

Researchers have traditionally shirked away from drawing as a behavioral measure because of its high level of variability: drawings can seem highly subjective, drawings are influenced by strategy and technique, and drawings are highly influenced by the drawing abilities of the participants. However, with enough participants and/or well-selected control conditions, these levels of variability can be easily washed out. In fact, so far all of our drawing studies have recruited from non-artists, with many participants expressing a lack of confidence in their drawing abilities.

The main crux of a successful drawing experiment is a bipartite structure: 1) a drawing task with careful control conditions, and 2) a battery of online scoring experiments to quantify the drawings. In Bainbridge et al. (2019), alongside having participants draw images from memory, a separate set of participants drew from the image (copying it), and a set of participants drew from a label of the image category (e.g., living room). Drawings copied from an image can serve as an *upper-bound*—few people will draw every pixel in an image, so what is drawn during perception serves as the maximum level of information one can expect to be drawn from memory. This upper bound also serves as a control for drawing ability; equal variability in drawing skill should be captured during both perception and memory. So when assessing memory drawings, the key question is how they compare to perceptual drawings, not how they compare to the original image. Drawings made from the name of an image category serve as a *lower-bound*, to demonstrate what information could be accurately drawn by solely remembering a semantic label for the image. For our memory task, we observed that participants recalled high levels of detail beyond this category representation; they did not draw just *any* living room, they drew the specific image of a living room that they studied (Bainbridge et al., 2019). This sort of task could also be used to understand people’s developments of schemas or concepts. Thus, carefully selected control tasks can manage the level of variability present in the condition of interest. When designing your own drawing experiments, it is essential to consider: what are your lower-bound and upper-bound measures (or, what are your controls)? Will you compare drawings to the original image, to drawings by another person, or within-participant drawings of another form? For example, in our study of aphantasia (Bainbridge, Pounder, et al., 2021a), we compared aphantasic memory drawings to same-participant perceptual drawings (within-subjects), but we also compared aphantasic memory drawings to control participant memory drawings (between-subjects). Having the same participant draw an image from memory and from perception can give a powerful measure of how their performance shifts from one process to the other. We also frequently compare drawings for a given image across participants, testing whether one condition or one group draws more detail than the other for the same image.

After obtaining these drawings, the next key point is that anything that can be observed in the drawings can be objectively quantified online. While classic drawing tasks require interpretation of the drawing by the experimenter or a clinician (e.g., Wechsler, 2009), in this era of online citizen science, scoring of these drawings can be crowd-sourced. This removes any subjectivity with interpreting the drawings, and the ease of crowd-sourcing allows for a large number of creative measures to be quantified. For example, one could measure the presence or location of a specific object, the viewing angle of the drawing, its mood or aesthetic qualities, etc. These online scoring experiments will be discussed in more detail later, with a list of properties that have been quantified from drawings thus far.

After solving these potential issues of subjectivity or drawing ability, we are left with an incredible gem of information—a visual mental representation. These drawings reveal how people see the world, with rich visual, semantic, and spatial information. While an individual drawing may be difficult to interpret, combining drawings for a given image, task, or participant creates a compelling picture book of underlying cognitive processes. These drawings can reveal not only memories, but concepts, interpretations, schemas, imaginings, and dreams. In this section, I describe how to collect drawings both in-lab and online, and discuss important considerations for designing these experiments.

## Drawing as measured in the laboratory

**Fig. 1:**
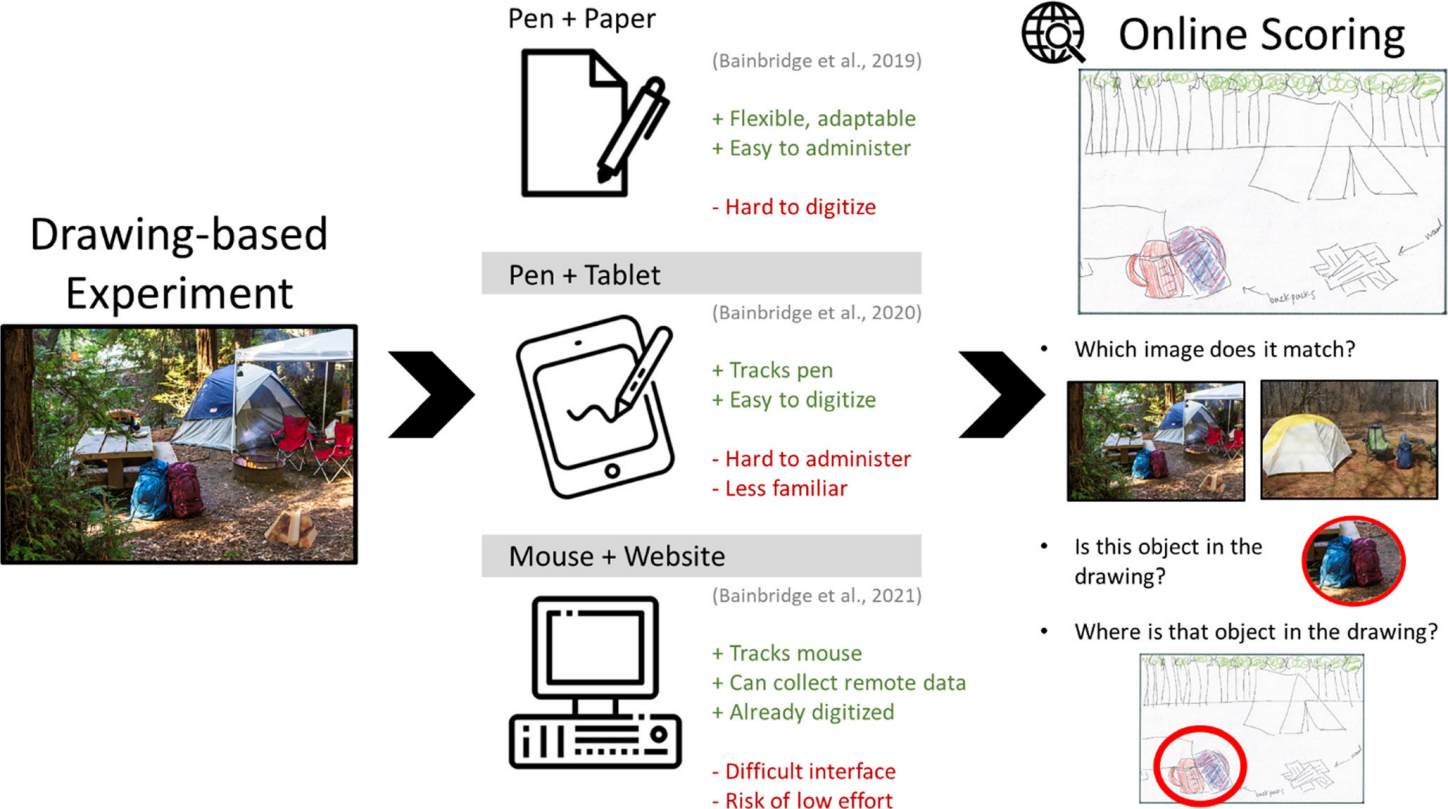
A broad flow-chart of the general methods of drawing experiments. First, participants view (or imagine) images in an experiment. The task and participant sample can vary flexibly—the only requirement is that the task require participants to draw. Then, participants create drawings, with one of three different interface options: a pen and paper, a pen and tablet, or mouse on a website. Some of the key pros (green pluses) and cons (red minuses) of each approach are listed here. Finally, these drawings are uploaded to an online crowd-sourcing platform where large numbers of online scorers judge these drawings for a range of fine-grained details

Drawing is an incredibly versatile methodology for in-person research, and adaptable to different experimental needs (Fig. 1). In its simplest form, it only requires paper and a pen, and is easily understood by most people. Because of its simplicity, a drawing task can be administered to a patient group, a classroom of students, and in different social groups. Conversely, one can also design a more complex and well-controlled study for in-lab participation.

In some of our recent drawing studies (Bainbridge, Kwok, & Baker, 2021b; Hall et al., 2021), in-lab participants studied images on a computer at a fixed visual angle while an eye-tracker (an EyeLink 1000 Plus) recorded their fixation patterns. They then drew each image from memory on a piece of paper with a rectangular border matching the size and dimensions of the original image. Importantly, they drew using a pen-tracking tablet (Wacom Intuos Pro Paper) so that we could capture pen movement patterns, and assess their link to eye movement patterns. Through pen tracking, we can measure many types of information, such as: time spent on specific details, order of drawing information, action trajectories reflecting unconscious processes in decision-making (Song & Nakayama, 2009), and errors. For example, we observed erasing behavior (reflected in pen movements but not the final drawing) and found that those with aphantasia show less editing than those with typical imagery (Bainbridge et al., 2021a, b).

One could envision modifying such a drawing task so it could be run simultaneously with neuroimaging. A temporally resolved method such as electroencephalography (EEG) or magnetoencephalography (MEG) could be utilized to link changes in brain activity to the time course of drawing (e.g., can we decode what is being drawn at a given moment?). Thus far, EEG research has shown alpha band activity patterns during drawing that could suggest improved learning (Belkofer et al., 2014; van der Meer & van der Weel, 2017). While slower in time scale, functional magnetic resonance imaging (fMRI) could be used to examine questions about inter-subject correlations during recall, or representations of information at a coarser time scale, adopting methods used to analyze verbal recall during fMRI (Chen et al., 2017). With MRI-compatible touchscreen interfaces (e.g., MRItab: Vinci-Booher et al., 2018), drawing can be natural and seamless inside an MRI scanner. Indeed, some labs have already identified brain networks engaged during drawing tasks (Gowen & Miall, 2007; Schaer et al., 2012), and compared representations during object drawing and object recognition (Fan et al., 2020). Additionally, experiments testing letter drawing during fMRI have revealed visual-motor networks of letter recognition (James & Gauthier, 2006; Vinci-Booher et al., 2019). Thus, drawing inside a scanner is certainly feasible, and could present a new way in which to decode mental representations.

## Drawing as measured digitally

**Fig. 2:**
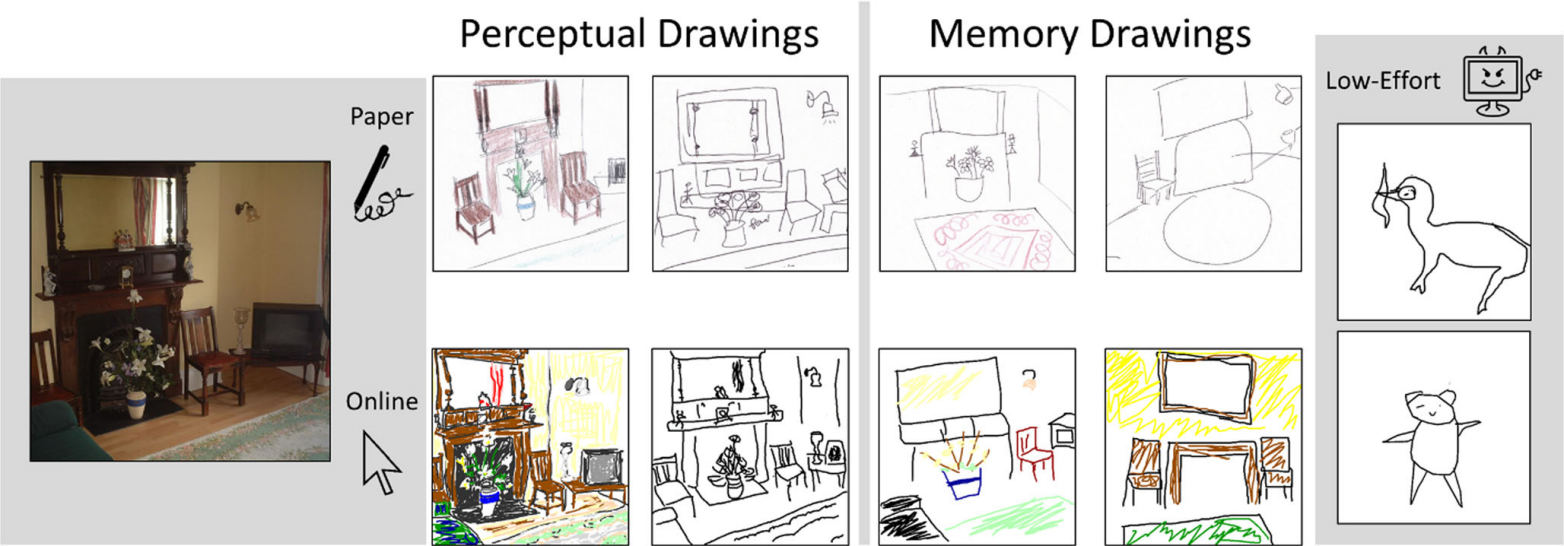
Example drawings from experimental data of the same living room photograph. Shown are example drawings all taken from separate participants, where they drew the same living room while viewing the image (“Perceptual Drawings”) or while recalling the image (“Memory Drawings”). Drawing quality was high regardless of whether participants were drawing with pen on paper (from Bainbridge et al., 2019), or with a mouse on a website (from Bainbridge, Pounder, et al., 2021a). It is also readily apparent when participants are not taking the task seriously or missed the stimulus, as seen from poor attempts at drawing the living room from two online participants (“Low-Effort”, from Greenberg & Bainbridge, 2021)

Online research is becoming increasingly popular in the psychological sciences, and offers a method to efficiently capture large amounts of data from diverse groups. For example, even though the prevalence of aphantasia is around 1–3%, we were able to recruit 61 online participants through Reddit and Facebook forums (Bainbridge, Pounder, et al., 2021a). The growing popularity of pen-based tablet devices also means that many online participants may have a comfortable way to draw digitally. However, even using an average computer mouse, participants can produce high-quality drawings in a web interface (Fig. 2).

In terms of implementation, we conduct our online drawing experiments using in-house code written in HTML and JavaScript (along with jQuery), available on the Open Science Framework page with this tutorial (https://osf.io/tgavx/). Coding without the need of proprietary software makes the code incredibly flexible and adaptable (and can be edited in a standard text editor). For drawing, we adapt an open-source jQuery plugin called wPaint (Websanova, 2011). This plugin works like a standard drawing program, where the participant can use a pen tool in different colors to draw lines, and an eraser to remove those lines. There are also undo, redo, and clear buttons to let the user fix any mistakes. While we thus far have only allowed the pen tool in our studies (, wPaint by default includes a wide range of tools, such as lines, text, ellipses, rectangles, and a fill tool. These tools could be useful to the experimenter, but in some cases the experimenter may want to remove extraneous tools to reduce the participant degrees of freedom. Once the drawing is complete, the image can then be saved as text using *base64* encoding. Most common programming languages can flexibly convert between an image format (like JPEG) and base64 (see code). We also use jQuery or JavaScript (a combination of its *mouseup()*, *mousedown()*, and *mousemove()* functions) to track the location and timing of the mouse while drawing. This results in output much like the in-lab pen tablet experiments, where you know what stroke is being drawn by the participant at any time. You can also track the movements they make before undoing or clearing data, which can capture drawing errors, or navigations away from the task.

**Fig. 3:**
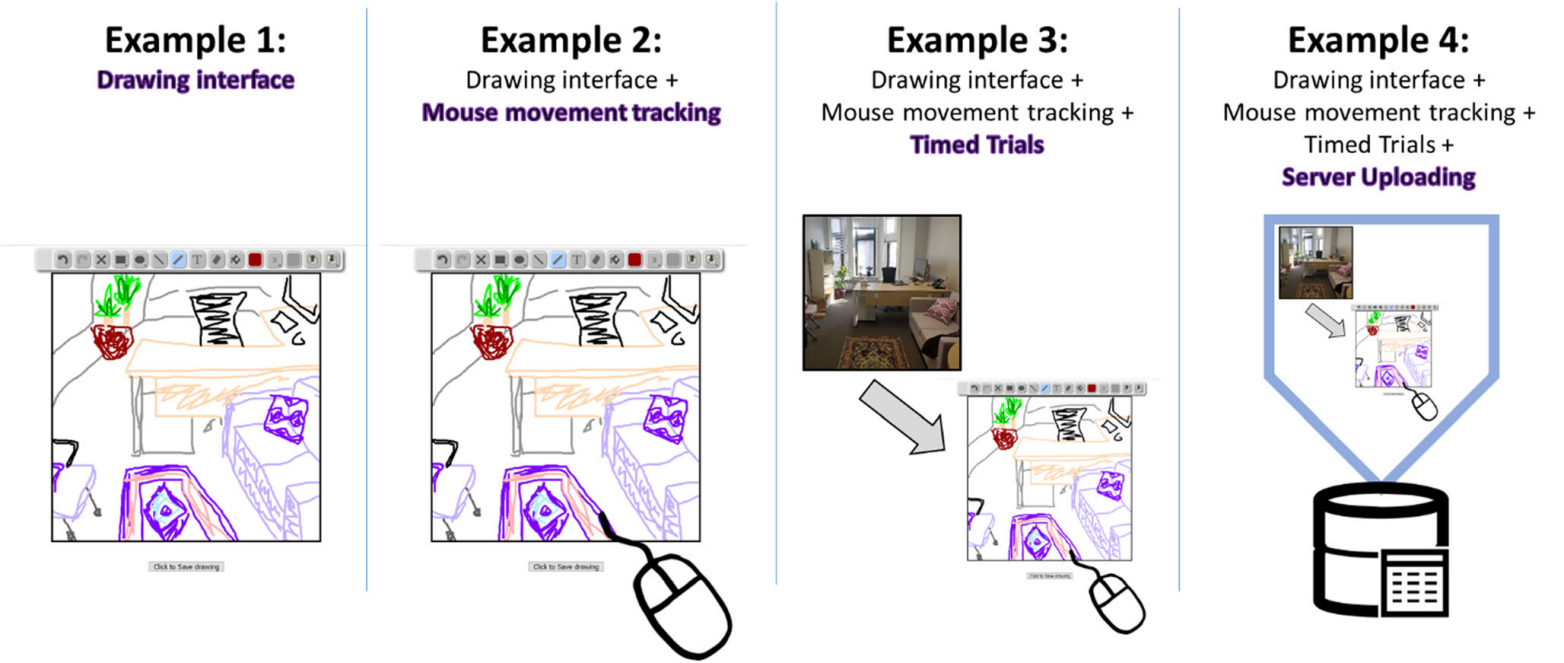
The four online drawing experiment examples available with this tutorial. The code is designed to provide examples at different levels of complexity, starting from a basic drawing interface (Example 1), and building up to a full-fledged experiment with timed trials, mouse tracking, and saving to a private server (Example 4). These examples could also serve as an iterative approach to learning web design, starting with basic HTML and JavaScript, building up to more complex JavaScript functions, and finishing with server-side scripting in PHP

Attached to this tutorial, we include code examples for four drawing experiments with increasing complexity, designed so that the code can be flexibly adapted for the reader’s uses, and also designed to teach some basic principles about web programming and architecture (Fig. 3). Example 1 includes the code for the simplest drawing interface, with HTML forming a basic page structure, and JavaScript used to load in the drawing interface and save the drawing. Building from Example 1, Example 2 adds JavaScript code that tracks and saves the mouse movements of the user. Example 3 provides an example of how multiple drawing interfaces can be integrated into a timed experiment with multiple trials. Finally, Example 4 shows how to integrate this code with a PHP script to save data to a private server.

When running an online experiment, there are some specific decisions one must make that are less important for in-person experiments. First, with a plethora of programming and online platform options available, it can be difficult to choose the right online host for any experiment. I discuss recommendations and considerations applicable to online experiments in Supplemental Information S1 (https://osf.io/q2vwz/). Second, online research is often plagued with an increased concern over data quality, because of the emergence of bots and task farms (Chmielewski & Kucker, 2019). Conveniently, it is usually relatively straightforward to identify low effort in drawings, and bots cannot yet perform these tasks. In Supplemental Information S2 (https://osf.io/q2vwz/), I provide targeted advice on avoiding cheating in online drawing tasks.

## Considerations when designing a task

### Task design decisions

While the implementation of a drawing task can be flexible, there are various decisions an experimenter must consider (Table 1). In our prior studies, we have never imposed a time limit on drawing time, however a time limit could be useful (e.g., to only capture top-priority items in one’s representations for an image). We often *do not* tell participants that they will be tested with drawing until they get to the drawing portion of the experiment (Bainbridge et al., 2019). We take this approach to limit drawing-targeted encoding strategies by participants, such as focusing on remembering information they know they will be better at drawing. We also test participants in a free recall manner, to avoid a risk of participants inserting false information based on cued information (e.g., if you tell them to “draw the living room” they studied, they may insert canonical living room objects—like a couch—even if they don’t specifically remember one). However, future experiments could intentionally manipulate task instructions or cues to see the role of strategies on memory performance. One final consideration is how long a delay the experimenter wants between presentation of the original image and the drawing (if any). Drawings take much longer than other behavioral outputs, with participants generally taking 2 min per drawing when given no time constraints (Bainbridge et al., 2019). Thus, memory for information might decay during the drawing period itself, and detail could dissipate with later drawings. We actually did not observe evidence for this in our original study; participants drew on average 12 images from memory and there was no evidence that later drawings contained less detail than earlier drawings (Bainbridge et al., 2019). However, there was a loss of detail when drawing after an intervening 12-minute distractor task in comparison to immediate recall. Thus, timing may be an important consideration, and one could use pen movements to quantify what information is recalled first or last.

**Table. 1:** Important considerations when designing a drawing experiment

Experimental design	Output measures
*Specific to in-lab studies:*	*Specific to in-lab studies:*
• What visual angle, resolution, and size will my images be? (Ensure drawing area matches this.)• Will I (the experimenter) watch the drawing process (and how might this influence participants)?*Specific to online studies:*• Will I restrict the hardware that can be used with the experiment (e.g., phone, tablet, monitor)?• Will I include catch trials or questions to ensure high data quality?*General considerations:*• How long will images be displayed for, and what fixation behavior will be allowed?• When will participants know they will be drawing? (At the beginning of the study? Right before the drawing portion?)• What instructions/cues will I use for each drawing trial? (Or will I make it a purely free recall task?)	• Will I record eye-tracking during the study phase?*Specific to online studies:*• Will I restrict the response devices that can be used (e.g., finger, stylus, mouse, joystick)?*General considerations:*• Will I record pen-/mouse-tracking during the recall phase? (And what measures do I care about? Time/stroke order? Speed? Pressure? Trajectory?)• Will I allow for color and/or text?• How long will participants be allowed to draw for?• Will I track erasures in some way?• What demographic information do I want to record? (e.g., artistic ability)

### Additional information beyond the drawing

Another consideration is whether participants should be able to include additional information beyond the line drawing. We have provided participants with colored pencils, and found that individuals with aphantasia use less color than those with typical imagery (Bainbridge, Pounder, et al., 2021a). We also allow participants to write text labels when they are unable to recall details, or are unconfident about their drawing for an object. These text labels can help an experimenter score or interpret an image (Bainbridge, Kwok, & Baker, 2021b), and have also revealed that individuals with aphantasia rely on semantic representations to scaffold their memory for an image (Bainbridge, Pounder, et al., 2021a). We also sometimes conclude experiments with a task where participants can indicate what image and objects they were intending to draw, to make scoring more straightforward (Hall et al., 2021). One could envision other types of information that could be collected in combination with a drawing. For example, experimenters could record participants verbally describing their drawing as they create it. Other sensor measurements such as pen speed or pressure could reflect unconscious cognitive processes, such as confidence or task difficulty (Song & Nakayama, 2009). Outside of the drawing task, I recommend collecting basic demographic questions about artistic experience (i.e., years of artistic training, ratings of one’s own drawing ability, occupation) in order to quantify individual variability in performance. We used these measures to demonstrate that individuals with aphantasia showed memory-specific deficits in their drawings, not explained by differences in general artistic ability (Bainbridge, Pounder, et al., 2021a).

### Limitations in drawing tasks

While drawings are information-rich, there are also limitations to drawing as a behavioral measure. First, drawings can be laborious to create, so the experimenter is limited in the number of drawings they can request before the participant is fatigued or out of time (the most we have requested is about 30: Bainbridge et al., 2019). With online experiments, the amount of dedicated focus the experimenter can expect is probably even shorter. Some individuals may also feel resistant to drawing—embarrassed about their abilities, or unsure where to begin. Finally, drawing ability can still be a barrier to accurately representing one’s mental representations; for example, one may have a clear image of a face but still be unable to draw it. Nonetheless, drawings can often capture more vivid visual details than other methods such as verbal report.

## Objective quantification of drawings

After collecting drawing data, the next important step is to quantify these drawings. Unlike classic drawing studies where the experimenter must use their discretion to score each drawing, a key innovation with this method is that the experimenter must outsource quantification of these drawings. In this way, we can leverage the rich ability of people to extract information from ambiguous input (a drawing), but utilize this ability at a large scale, unbiased by the experimental questions, to create objective scores for each drawing. Table 2 lists a survey of the types of measures that can be captured from drawings. Using human ratings, one can quantify a range of information from image-based metrics, to detail-level metrics analyzing specific portions of an image. Computationally derived measures can also be directly compared with these human metrics. This tutorial includes a base of code to create, test, and analyze online scoring experiments for drawings, as well as compare their results to computationally derived measures.

**Table. 2:** Examples of information that can be quantified from drawings

Image-level metrics	Detail-level metrics	Computational metrics
Image/scene identity	Object identities	Saliency
Drawing quality	Feature size/location/orientation	Color/luminance
Aesthetic quality	Similarity to a schema/exemplar	Symmetry
Emotional valence	Texture and material of objects	Spatial frequency, edges, GIST
Viewing perspective	Caricaturization of features	Mid-level measures: SIFT, HOG
Realism/imaginativeness	Usage of color	Deep learning: classification & heatmaps
Similarity to a schema	Usage of text labeling	Meaning maps
Accuracy	Drawing order & time	Motion maps
Erasures and errors	Object-object relationships	Semantic segmentation
Interpretability	Insertions of false details	Generative Adversarial Network inputs

### Image-level metrics

**Fig. 4:**
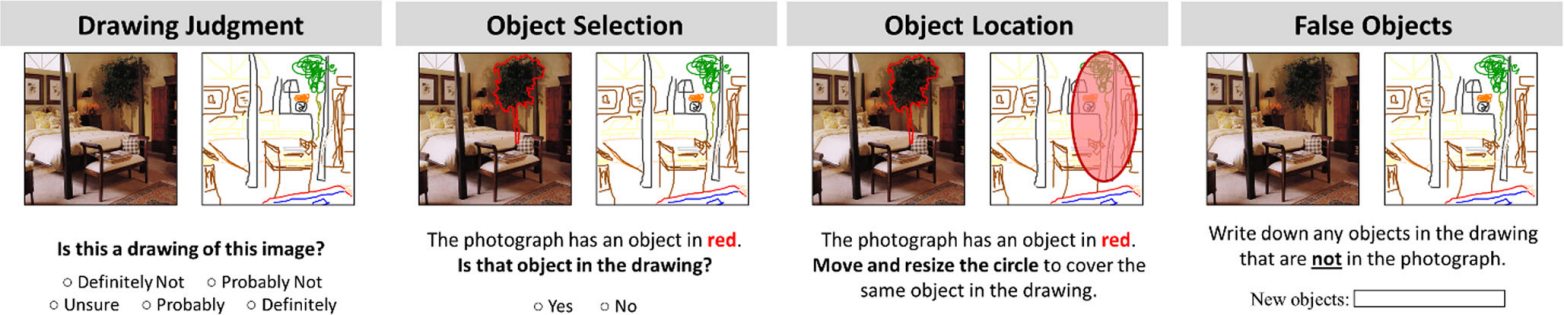
Example online scoring experiments for the drawings. One can use online scoring to collect image-level metrics, such as how well a drawing matches an image (“Drawing Judgment”) or what differences may exist between the drawing and image (“False Objects”). Online scoring can also give fine-grained detail-level metrics, with online participants providing a response for every object, such as whether each object exists in a drawing (“Object Selection”) or where it is located and at what size (“Object Location”). These four specific experiments have been validated across studies (Bainbridge et al., 2019; Bainbridge, Kwok, & Baker, 2021b; Bainbridge, Pounder, et al., 2021a; Hall et al., 2021), and are provided in the code base of this tutorial

The most straightforward of metrics to crowd-source are those where online scorers must make a singular judgment of a drawing (Fig. 4). Viewing a drawing, a scorer could be asked to make a range of responses along a Likert scale, for example scoring the drawing quality, aesthetic quality, emotional valence, realism, interpretability, or other similar measures. They can also be asked to compare the drawing to another image or set of images, for example judging: How similar is this drawing to this image?; Which viewing perspective does this drawing take (e.g., front, ¾, side, birds-eye)?; How similar is this drawing to the average schema across these photographs? Online scorers can also be asked to make short responses related to the drawing, such as: What is this a drawing of?; Name the objects in this drawing; How would you describe this drawing to someone? Finally, online scorers can also make judgments based on a video of the mouse movements of a drawing, labeling features such as the existence of erasures/editing, the order of pen strokes, or the speed of drawing.

### Detail-level metrics

Perhaps most interesting and innovative of the described approach is to rely heavily on detailed scoring of images. A first principle is that many of our experiments are *automatically derived*. For example, one common experiment is having online scorers judge which objects from a photograph exist in a drawing of that photograph (Fig. 4). To create this experiment, we first identify the outlines of all of the objects in the photograph. This can be conducted by someone in-lab or through crowd-sourcing using LabelMe (http://labelme.csail.mit.edu/Release3.0/, Russell et al., 2007), an online interface where you define the polygons that form the outlines of each object in an image. LabelMe saves these annotations as an XML file defining the coordinates of each object polygon’s points, which serves as a file that can be flexibly read into many programming languages. Similar outlines could also be derived using a computer vision or deep learning algorithm such as VGG-16 for object classification (Simonyan & Zisserman, 2014). With these outlines, we know what objects are in an image, at what size, and what location. We can create (and have provided) code that can automatically create one image for every object, in which its outline is highlighted in red in the context of the scene. We then automatically create thousands of online scoring trials where a scorer sees a drawing and an object highlighted in red and has to respond whether that object is in the drawing or not. These ratings are collected across a number of scorers (we typically use five per object, given the large number of objects we collect judgments on), and then the majority response wins, to determine what objects exist in a drawing.

A second principle is that we run multiple *nested experiments*, where the trials of one experiment are derived from the trials of the previous experiment. For example, another possible experiment is to derive the locations of every object in a drawing (Fig. 4). To do this, we automatically create online scoring trials only for the objects that are judged to exist in a drawing in the task described previously. For those specific trials, we show online scorers the photograph with an object highlighted in red, and the matching drawing with a superimposed red ellipse. The job of the participant is to move and resize the ellipse to highlight that corresponding object in the drawing. We collect five ellipses per object, and take the median center and radii as the final ellipse for the object. Across all of our studies, these measures have indicated a high spatial accuracy, where objects tend to be drawn at the same sizes and locations as the original objects in the photographs (Bainbridge et al., 2019; Bainbridge et al., 2021a, b), suggesting this ellipse measure is successful. Future versions of this experiment could use a LabelMe-like interface to instead measure a complex contour for a drawn object.

After these object identification and object location experiments are complete, you now have a direct correspondence of objects in the drawing to objects in the photograph. This allows the experimenter to ask a wide range of questions. You can examine how usage of detail or color varies within and across individual objects. You can examine what task manipulations modulate the drawing-photograph correspondences (for example: does a longer memory delay reduce the spatial correspondence between the two?). You can investigate when an object was drawn and for how long, just as you can measure when and how long an object was fixated on in a photograph. You can ask online scorers to judge individually drawn objects in ways they can judge the entire image: scoring an object for aesthetic or emotional value, or describing it or attributing it a label. You can apply similar methods not just to singular objects, but also to clusters of objects, object parts, or features (e.g., the features in a face). Finally, you can look at false memories – what objects are drawn that do not exist in an original image, and where are they?

**Fig. 5:**
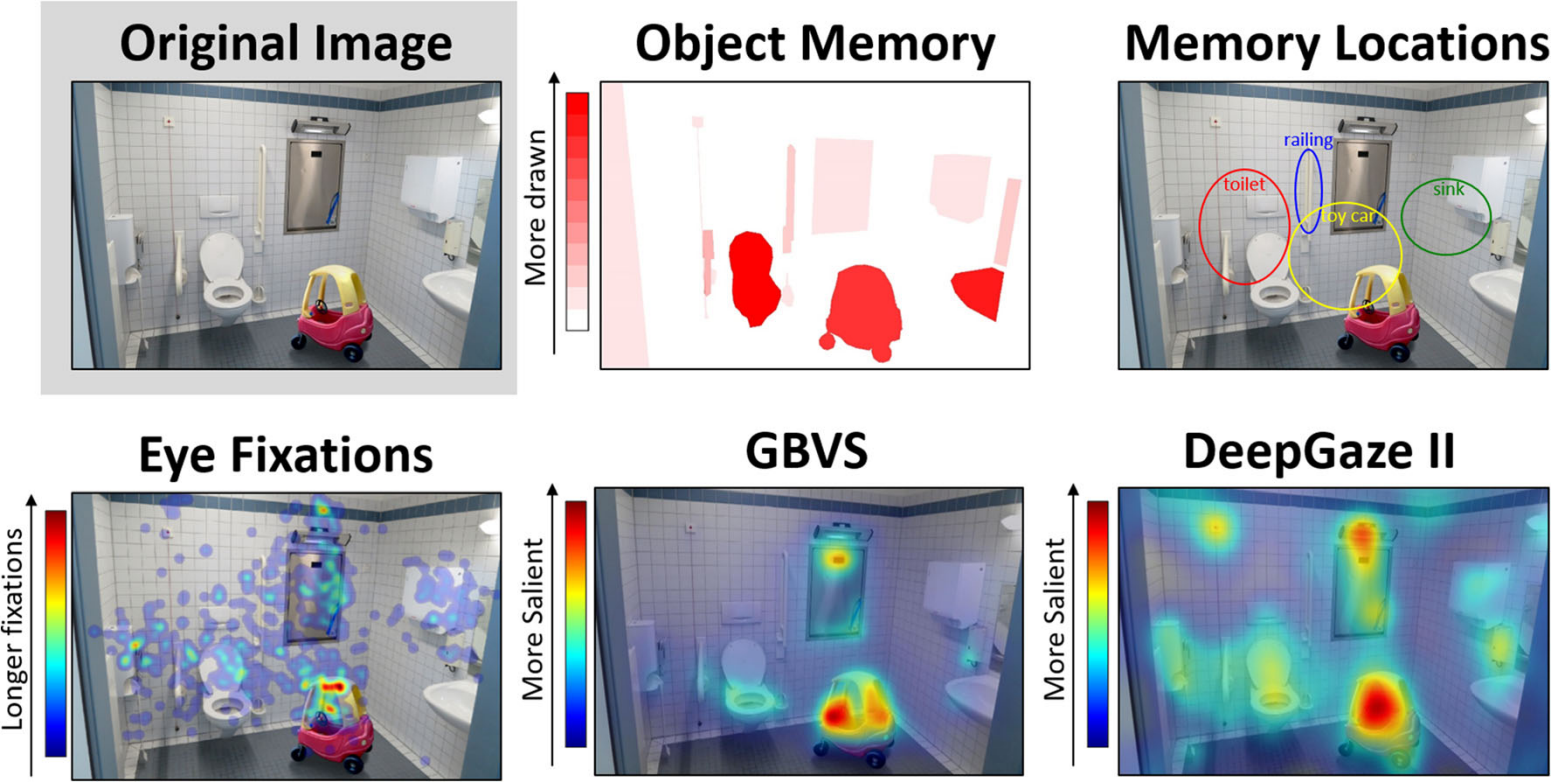
Different quantifications of the same image. Here we illustrate how different human- and computationally derived metrics can be compared in the pixel or object space between images and drawings. From the *Object Selection* online experiment, one can create a heatmap indicating the proportion of participants who drew each object (“Object Memory”). One can also use the *Object Location* online experiment to derive the locations of drawn objects (“Memory Locations”, showing the median ellipse for the top four drawn objects). These object values and locations can then be compared to the values and locations of participants’ fixation behavior on the image (“Eye Fixations”), or computer-derived image metrics like Graph-Based Visual Saliency (“GBVS”) or DeepGaze II neural network predictions (“DeepGaze II”). The original photograph and example data come from Bainbridge, Kwok, & Baker, 2021b

This image-object-to-drawn-object correspondence also means that drawings and images can be transformed into representations defined by the object space. It may be difficult to compare drawings and photographs at the pixel level because drawings tend to be sparser across their pixels (although see the next section). Instead, drawings and photographs can be compared at the object level, where a given image or drawing is represented by a vector of objects. For example, you could compare a vector of fixation durations on objects in a photograph and a vector of drawing durations on objects in a drawing. As another example, you could compare ratings of aesthetic values of objects in a photograph to ratings of drawing quality of objects in a drawing. The same methods could also be used for other types of discrete elements, for example to compare the locations of facial features in a photograph to the locations of facial features in a drawing. Conceptualizing images and drawings in an object space also allows one to perform computations across drawings – for example, looking at the average aesthetic rating across individuals for a given drawn object, or collecting a proportion of people who drew a given object. This allows for the creation of object-based heatmaps that can visualize these values using the stable locations of the objects in the original image, even when showing data from the drawings (Fig. 5).

### Computationally derived metrics

Many measures that can be computationally derived from a photograph can be computationally derived from a drawing as well. These algorithms can vary in complexity by pixel-level computations, more complex image-based metrics, and deep-learning-derived metrics.

First, many measures can be derived through directly examining the pixels of the drawing. By averaging the pixel values at each color channel, one can get an idea of how much information is present for each basic color (R, G, B), for example, how blue the drawing is. By instead measuring the variance, one captures a measure of the contrast in the image. If drawings are made in an online interface, the experimenter can limit the specific colors that can be used in the interface, and the number of pixels matching each color can be quantified to provide a fine-grained measurement of the distribution of colors. The density or amount of “ink” in the drawing can be quantified as the total number of non-white pixels. From the pixels of an image, one can also measure symmetry and spatial frequency. To quantify symmetry, one can divide the image into two halves and subtract one half with the mirror-flipped version of the other. The more non-zero pixels that remain, the more asymmetric the image is. Finally, spatial frequency captures the edge information or spectral energy present in the image, and can be calculated by looking at the Fourier transform of the drawing. The Natural Image Statistical Toolbox is an open-source MATLAB toolbox that can quantify these measures of color, contrast, symmetry, and spatial frequency (Bainbridge & Oliva, 2015).

Many tools developed in the realm of computer vision already exist for quantifying higher-level information about images, such as HOG, SIFT, and GIST. HOG (histogram of oriented gradients) is an image feature often used for object classification that analyzes edge orientations in local image regions (Dalal & Triggs, 2005). SIFT (scale-invariant feature transform) is a feature often used for image alignment that compares local regions of an image to a reference set (Lowe, 1999). Finally, GIST captures the gist and spatial envelope presented by the spectral energy within an image (Oliva & Torralba, 2001). These features can be used to parse the content of an image, for example, they can be used to automatically classify the genre of a painting (Agarwal et al., 2015). However, they have not been used in the field of psychology as metrics to quantify drawings, although there are toolboxes available for quantifying images (Khosla, 2017). Visual saliency is also a common metric that can be quantified using tools such as Graph-Based Visual Saliency (GBVS, Harel et al., 2007). GBVS creates a heatmap of the salient portions of an image (in other words, the parts of an image that are most visually dissimilar from other parts), and can predict eye movement patterns.

More recently, deep learning neural networks (DNNs) are becoming increasingly common ways to quantify an image. They serve as compelling models of the early human visual system (Cichy et al., 2016; Yamins et al., 2014), and have made strides in predicting object perception through networks like AlexNet (Krizhevsky et al., 2012) and VGG-16 (Simonyan & Zisserman, 2014), scene perception through networks like Places-CNN (Zhou et al., 2014), fixations through networks like DeepGaze II (Kümmerer et al., 2016), and memory through networks like MemNet (Khosla et al., 2015) and ResMem (Needell & Bainbridge, 2021). Most of these pre-trained networks are publicly available, and can be used to make inferences about novel images. For example, one could measure: To what degree can the objects or scene of a drawing be classified? How does memorability of a drawing compare to memorability of the original image? Do predicted fixations on an image relate to what objects people will draw? Some DNNs also create heatmaps across the image, such as MemNet, which creates a heatmap of what image information most contributes to its predicted memorability score. There has also been burgeoning work in relating DNN-based representations of drawings to those of the corresponding image (Fan et al., 2018) and using DNNs to score drawing recognizability (Long et al., 2021).

There are two main ways in which these computational methods can be used to quantify drawings. Most directly, they can be used to quantify both the drawing and its original photograph, and then those two can be directly compared. For example, a singular value of color contrast could be derived for each photograph and each drawing and then correlated. For another example, a GBVS heatmap could be created for a photograph and a GBVS heatmap could be created for a drawing, and these saliency measures could be correlated pixel-by-pixel. However, drawings will intrinsically differ on many visual features in comparison to photographs, and often not for particularly interesting reasons. For example, drawings likely will include less color across all the pixels than a photograph, since it is effortful and inefficient to color each pixel. There also may not be as many fine details within a drawing and lines may not be as straight, thus influencing spatial frequency and symmetry measures. Instead, it may make more sense to transform these measures to the object space. For example, one could look at the color contrast of each object, normalized by the mean color contrast across objects, and compare this measure between the photograph and a drawing. Through this normalization, you would remove the differential influences of color usage in drawing versus an image. For heatmap measures like GBVS, you can compute the average value across the pixels contained within an object (e.g., the average saliency of the chair), and compare it to another measure (e.g., the proportion of people who drew that chair). Putting these computational metrics into the object space also makes it easier to combine data across drawings of the same image.

## Applications of this method to other questions and fields

While this drawing method has thus far been applied to questions related to memory and visual imagery, this method has large-reaching implications across questions in psychology. Here I will provide some launching points for ideas in alternate subdomains of psychology and neuroscience, although I envision the number of possible questions is infinite.

This method can serve as a tool for understanding schemas or concepts. Drawing has been used to investigate the emergence of schemas in children (Freeman & Janikoun, 1972; Long et al., 2021), the influence of culture on schemas (Axia et al., 1998), and to question whether children even can use drawings to represent schemas (Kosslyn et al., 1977). Drawings have also revealed how experts differentially organize information through chunking (e.g., investigating electronic technicians’ memories for circuit diagrams: Egan & Schwartz, 1979). Online crowd-sourced scoring and digitized drawings can allow for even more fine-grained views into category schemas and the role of expertise across the lifespan.

Drawings can be used to capture current cognitive states and their fluctuations within an individual. Just as emotion and stress can color our memories or perception, they likely color our drawings as well. In fact, drawing is occasionally used as a method for children to indicate emotional states (Thomas & Jolley, 1998) and moments of stress (Rollins, 2005). Regardless of age, the content and nature of one’s drawings may shift with changes in emotional state or stress, and these drawings could be useful as diagnostic or communicative tools. For example, what errors may be introduced in a drawing (e.g., false additional items, missing items) during times of stress? How do anxiety or depression influence the features of a drawing? And, can emotional state be predicted from drawings, even when made from images without an inherent emotion?

The act of drawing itself may also have a direct influence on cognitive abilities, informing questions on the plasticity of the brain. Artists, who are highly trained at drawing, show different fixation patterns from non-artists when viewing an image, and recall more details (Vogt & Magnussen, 2007). Even for non-artists, drawing when learning information shows larger benefits over verbally rehearsing information in adults (Perdreau & Cavanagh, 2016; Wammes et al., 2016) and children (Gross & Hayne, 1998). However, drawing can also inflate the existence of falsely recalled information (Bruck et al., 2000; Otgaar et al., 2016). Future studies could examine how artistic training influences the accuracy of drawings, and translates to performance in other non-drawing tasks. Drawing also acts as an interaction of multiple processes: perception, attention, and motor control (Cohen & Bennett, 1997; Makuuchi et al., 2003). The highly quantified drawings proposed here can investigate how subtle manipulations in perception, integration, attention, and motor ability influence the content of drawings.

Drawings also can reveal insight into higher-level questions about social interactions, decision making, and morality. Children draw more accurately when their drawings are used as a form of social communication (Light & McEwen, 1987). Children also draw family members differently based on their attachment style (Goldner & Scharf, 2011). Thus, there are clear social influences on drawing even from an early age. Along a similar vein, what differences might emerge when drawing people from different social categories? In a study by Uddenberg and Scholl (2018), when white participants reconstructed a face from memory utilizing an interface that generates virtual faces along a continuum, they tended to reproduce a face as more white that it was. Thus, reconstruction tasks like drawing could unveil subtle biases related to our representations and perceptions of social categories like race and gender. Drawings could also be used to indicate perceptions of different decision-making options or moral choices. For example, we often internalize an intrinsic value for objects, such as tastier foods having high value (Bakkour et al., 2019), and such value differences may appear in drawings of these objects.

Finally, while drawings of specific shapes are sometimes included in test batteries with patients (the Ray-Osterrich figure to test memory: Shin et al., 2006; the clock-drawing test for spatial neglect: Agrell & Dehlin, 1998), drawings of complex, naturalistic images promise a deeper look into variations in cognitive experience. I have already described work showing separate systems for object and spatial imagery in individuals with aphantasia (Bainbridge et al., 2021a, b). Aphasic patients (individuals with diminished language ability) also show marked deficits in drawing objects from memory, correlated with diminished language abilities (Gainotti et al., 1983). Performance on a clock drawing test can also differentiate healthy elderly adults from those with Alzheimer’s disease (Cahn et al., 1996). Thus, in many ways, deficits may be reflected in one’s drawings, as drawing requires an interwoven combination of many cognitive abilities.

## Conclusions

While drawing has often been regarded as a noisy task, delegated mostly to test batteries in the clinical domain and historic studies before the advent of computers, there is much untapped potential in drawing as a rich, informative behavioral measure. Leveraging modern methods of large-scale online experiments, crowd-sourcing, and high quantification through computer vision and pen-tracking, new drawing experiments have already revealed exciting insights about memory, perception, and attention, and promise to answer plentiful questions across psychology. This tutorial serves as a basis to equip any researcher—regardless of programming knowledge—with the tools to conduct high-quality drawing experiments, and be aware of the big picture questions one must keep in mind when running these studies. With this, we will be better able to draw out the mental representations in people.
